# Immunotherapies in Huntington's disease and α-Synucleinopathies

**DOI:** 10.3389/fimmu.2020.00337

**Published:** 2020-02-25

**Authors:** Oluwaseun Fatoba, Yosuke Ohtake, Takahide Itokazu, Toshihide Yamashita

**Affiliations:** ^1^Department of Molecular Neuroscience, Graduate School of Medicine, Osaka University, Suita, Japan; ^2^WPI -Immunology Frontier Research Center, Osaka University, Suita, Japan; ^3^Department of Neuro-Medical Science, Graduate School of Medicine, Osaka University, Suita, Japan

**Keywords:** Huntington's disease, α-synucleinopathies, immune activation, immunotherapy, combination therapy

## Abstract

Modulation of immune activation using immunotherapy has attracted considerable attention for many years as a potential therapeutic intervention for several inflammation-associated neurodegenerative diseases. However, the efficacy of single-target immunotherapy intervention has shown limited or no efficacy in alleviating disease burden and restoring functional capacity. Marked immune system activation and neuroinflammation are important features and prodromal signs in polyQ repeat disorders and α-synucleinopathies. This review describes the current status and future directions of immunotherapies in proteinopathy-induced neurodegeneration with emphasis on preclinical and clinical efficacies of several anti-inflammatory compounds and antibody-based therapies for the treatment of Huntington's disease and α-synucleinopathies. The review concludes with how disease modification and functional restoration could be achieved by using targeted multimodality therapy to target multiple factors.

## Highlights

- Modulation of immune activation in Huntington's disease (HD) and α-synucleinopathies using immune-based therapies appears promising.
- Several immune-based therapies failed to meet the clinical trial primary endpoint.
- Development of multimodality therapy that target neuroinflammation and other neurocircuitry disruptions might be warranted.

## Introduction

Nearly all major neurodegenerative diseases are proteinopathic in origin. Neurodegenerative proteinopathies are characterized by abnormal deposition of pathogenic protein aggregates in the form of inclusion bodies. Hereditary polyglutamine (polyQ) triplet disorders and α-synucleinopathies are classical examples of neurodegenerative proteinopathies marked by the accumulation of specific pathogenic protein aggregates in specific brain regions. Hereditary polyQ triplet disorders are rare, inherited forms of neurodegenerative diseases caused by the abnormal expansion of CAG trinucleotides repeats encoding a polyQ tract in the coding regions of specific genes within the genome. The consequences of the polyQ repeat expansions appear to be at the protein level, resulting from toxic gain, or change-of function mutations (1). Currently, nine disorders fall under this category of neurological diseases, including Huntington's disease (HD), spinobulbar muscular atrophy (SBMA), dentatorubral-pallidoluysian atrophy (DRPLA), and spinocerebellar ataxias (SCA1, SCA2, SCA3, SCA6, SCA7, and SCA17) (2–4). Among the hereditary polyQ triplet disorders, HD appears to be the most representative. HD evolves from an abnormal expansion of polyQ repeats in the huntingtin (Htt) gene. The resultant mutant Htt protein (mHtt) forms ubiquitously expressed intracellular aggregates throughout the body, particularly in the brain where it is cytotoxic to specific neurons in the striatum and cortex, causing neurotoxicity and devastating neurodegeneration. The pathological features of HD are summarized in Table 1. α-Synucleinopathies are common progressive neurodegenerative diseases caused by the abnormal formation of αsynuclein (α-Syn) and its aggregation within neuronal or glial cells (18). α-Syn related diseases include Parkinson's disease (PD), dementia with Lewy bodies (DLB), and multiple system atrophy (MSA). α-Syn abnormal deposition, which forms β-sheet enriched amyloid fibrils is a neuropathological hallmark of α-synucleinopathies (18). While these diseases share the same pathological protein, the pathological phenotypes from distinct α-synucleinopathies are quite different among patients (Table 1).

**Table 1:** Clinical features of HD and α-synucleinopathies.

**Disorder**	**Mutated gene**	**Pathogenic protein**	**Neuropathology and CNS region affected**	**Clinical manifestations**	**References**
**Inherited PolyQ repeat disorder**					
HD	IT-15 (HD gene)	mHtt	Neuronal intranuclear inclusions of mHtt in the striatum and cortex	Chorea, dystonia, bradykinesia, motor incoordination, psychiatric symptoms, cognitive decline, weight loss, skeletal muscle atrophy, sleep disorder, autonomic disturbance	(5–7)
**α-Synucleinopathies**					
PD	SNCA	α-Syn	LBs and LNs in the substantia nigra pars compacta dopaminergic neurons	Asymmetric bradykinesia, rigidity, unilateral resting tremor, cognitive deficits, rapid eye movement sleep disorders, pain, sensory deficits, GI motility disturbance, sialorrhea, and or- thostatism	(8–11)
DLB	SNCA	α-Syn	LBs LBs and LNs in the cortex and limbic system	Parkinsonism, early cognitive deficits (dementia), depression, sleep difficulties, attention deficit, and visual hallucination	(12, 13)
MSA	SNCA	α-Syn	GCI (Glial cytoplasmic inclusions) in the oligodendrocytes	Parkinsonism, dysautonomia, and motor dysfunction, cerebellar ataxia	(14–17)

*HD, Huntington's disease; PD, Parkinson's disease; DLB, dementia with Lewy bodies; MSA, multiple system atrophy; IT-15, interesting transcript 15; α-Syn, α-synuclein; SNCA, α-synuclein gene; LB, Lewy body; LN, Lewy neurite*.

**Figure 1:**
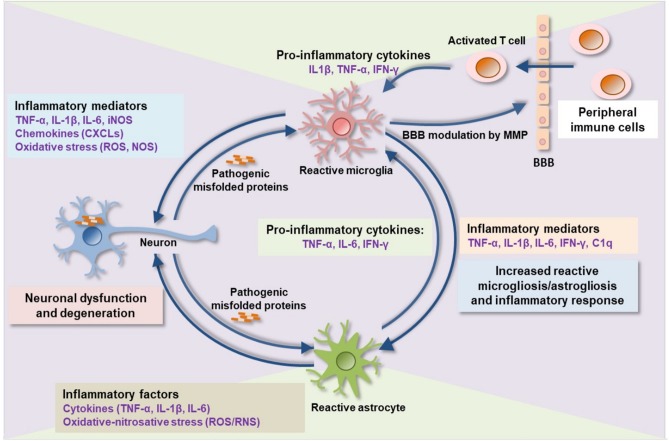
Proteinopathy-driven neuroinflammation. Pathogenic misfolded protein aggregates interrupt CNS immune homeostasis. Activated microglia release pro-inflammatory cytokines, chemokines, complement factors, nitric oxide, free radicals, and proteases that in turns mediate brain inflammation and consequent neuronal damage. Pro-inflammatory mediators such as MMP damages BBB integrity leading to an infiltration of peripheral immune cells (activated T-cells) into the brain which consequently activates microglia, astrocytes, and, neurons to release additional inflammatory molecules, thereby augmenting neuroinflammation and neurodegeneration. BBB, blood-brain barrier; MMP, matrix metalloproteinase; IL, interleukin; TNF-α, tumor necrosis factor-alpha; IFN-γ, interferon-gamma; C1q, complement component 1q; CXCLs, chemokine (C-X-C motif) ligand 1; iNOS, inducible nitric oxide synthase; ROS, reactive oxygen species; RNS, reactive nitrogen species.

A common pathological signature of neurodegenerative proteinopathies is chronic immune activation, encompassing innate and adaptive immune responses (Figure 1). Over the past decade, a plethora of studies has demonstrated a remarkable connection between pathogenic misfolded protein aggregates and immune activation in HD and α-synucleinopathies (Tables 2, 3).

**Table 2:** Important clinical and experimental findings on chronic immune activation in HD.

**Study (Reference)**	**Study purpose**	**Relevant findings**
Early and progressive accumulation of reactive micro-glia in the HD brain (19)	Investigation of localization of microglia in control and HD brain by immunohisto-chemistry	• Presence of activated microglia in the neostriatum, cortex, and globus pallidus, and the adjoining white matter of the HD brain • Reactive microglia contacted mHtt inclusion containing pyramidal neurons
Microglial activation correlates with severity in HD: a clinical and PET study (20)	*In vivo* evaluation of microglia activation at different stages of HD using PET imaging with microglia activation marker ([^11^C](R)-PK11195 PET) and D2R-bind-ing marker ([^11^C]raclopride PET)	• Significant increase in striatal [^11^C](R)-PK11195 binding in the prefrontal cortex, cingulate cortex and striatum. • Striatal [^11^C](R)-PK11195 level correlate with dis-ease severity assessed by the UHDRS motor score
A novel pathogenic pathway of immune activation detect-able before clinical onset in HD (21)	Examination of the relationship between peripheral immune activation and CNS pathology in HD	• Significant increase in plasma levels of IL-6, IL-8, IL-4, IL-10, and TNF-α in HD mouse models and patients • Increased IL-6, IL-8 and TNF-α in the HD CSF and postmortem HD striatal tissue • Remarkable correlation between plasma TNF-α levels and UHDRS chorea scores, motor scores, and total functional capacity • Monocytes, macrophages, and microglia from HD mouse models and patients express mHtt and dis-played functional over-activity when stimulated with LPS and IFN-γ
Mutant Htt promotes autonomous microglia activation via myeloid lineage-deter-mining factors (22)	Investigate whether mHtt expression alters microglia function in a cell-autonomous fashion using genome-wide approaches	• Expression of mHtt in microglia promoted cell autonomous pro-inflammatory transcriptional activation of the myeloid lineage-determining factors PU.1 and C/EBPs • mHtt-expressing microglia trigger neuronal apoptosis *ex-vivo* and *in-vivo*
Mutant Htt fragmentation in immune cells tracks HD progression (23)	Quantification of total Htt and mHtt in HD peripheral immune cells by TR-FRET immunoassay	• Increase mHtt expression levels in monocytes, T cells, and B cells HD patients • Monocyte and T cell mHtt levels were significantly associated with disease burden scores and caudate atrophy rates in HD patients
A critical role of astrocyte-mediated nuclear factor-kappa-B-dependent inflammation in HD (24)	Investigation of mechanism of astrocytic inflammation in HD	• Enhanced activation of NFκB-p65 activity in the astrocytes of HD patients and mouse models • Blockage of IKK ameliorates astrocyte-mediated NFκB-dependent inflammatory response and neuro-toxicity in HD R6/2 mouse model
HTT-lowering reverses Huntington's disease immune dysfunction caused by NFkappaB pathway dysregulation (25)	Identification of mechanism of dysfunction in primary human HD monocytes and macrophages *ex-vivo*	• Human HD myeloid cells produce excessive inflammatory cytokines as a result of the cell intrinsic effects of mutant huntingtin expression via NFκB signaling pathway
*In vivo* neutralization of the protagonist role of macro-phages during the chronic inflammatory stage of HD (26)	Assessment of HD mouse monocyte, macrophage, and other immune cells from blood, brain and/or spleen during early symptomatic and late stage HD	• Elevated plasma levels of IL-6, IL-10, and TNF-α • Increased striatal IL-12 and TNF-α mRNA transcripts • Elevated splenocyte IL-10, IL-12, and IL-17 mRNA transcripts

*C/EBPs, CCAAT-enhancer-binding proteins; PET, positron emission tomography; TR-FRET, time-resolved fluorescence energy transfer; D2R, dopamine D2 receptor; HD, Huntington's disease; Htt, Huntingtin; UHDRS, unified Huntington's disease rating scale; NFκB, nuclear factor kappa-light-chain-enhancer of activated B cells; IL, interleukin; TNF-α, tumor necrosis factor-alpha; IFN-γ, interferon gamma*.

**Table 3:** Important clinical and experimental findings on chronic immune activation in α-synucleinopathies.

**Study (Reference)**	**Study purpose**	**Relevant findings**
Microglial activation and dopamine terminal loss in early PD (27)	*In vivo* investigation of changes in microglial activity associated with changes in the presynaptic dopamine trans-porter density in the PD brain using PET imaging with microglia activation marker ([^11^C](R)-PK11195-PET) and dopamine transporter marker ([^11^C]CFT-PET)	• Increased midbrain [^11^C](R)-PK11195-PET-BP • Midbrain [^11^C](R)-PK11195-PET activity correlated inversely with [11C]CFT-BP in the putamen • Midbrain [^11^C](R)-PK11195-PET activity correlated with the motor severity assessed by the Unified Parkinson's Disease Rating Scale (UPDRS)
*In vivo* imaging of microglial activation with [^11^C](R)-PK11195 PET in idiopathic PD (28)	*In vivo* evaluation of brain distribution of activated microglia in idiopathic PD using ([^11^C](R)-PK11195-PET)	• Widespread microglia activation is associated with pathological processes in PD but did not correlate with clinical severity or putamen [^18^F]-dopa uptake
Peripheral cytokines profile in PD (29)	Investigation of levels of production and expression of cytokines and chemokines by PD patients-PBMCs	• Significant increase in basal and LPS-induced levels of MCP-1/CCL3, RANTES/CCL5, MIP-1α, IL-8, IFNγ, IL-1β and TNFα
Direct transfer of α-Syn from neuron to astroglia causes inflammatory responses in synucleinopathies (30)	Investigation of mechanism of glia inter-action and glial α-Syn pathology in α-Syn transgenic mice	• α-Syn released from neuronal cells are endocytosed by astrocytes through and form glial inclusions that triggers pro-inflammatory functionally polarized phenotype of astrocytes
α-Syn fibrils recruit peripheral immune cells in the rat brain prior to neurodegeneration (31)	*In vivo* assessment of MHCII-expression and neuroinflammation profiles in rat model of α-Syn-mediated neurodegeneration	• α-Syn fibrils promote microglial activation with peripheral immune cell infiltration in the SNpc α-Syn fibrils rapidly induce a persistent MHCII response derived from both microglia, monocytes and macrophages
Peripheral monocyte entry is required for α-Syn induced inflammation and neurodegeneration in a model of PD (32)	Investigation of peripheral monocytes in mouse model of α-Syn-mediated neurodegeneration	• Overexpression of α-Syn induces robust infiltration of pro-inflammatory CCR2-positive peripheral monocytes into the substantia nigra • Genetic deletion of CCR2 prevents α-Syn induced monocyte entry, attenuates MHCII expression, and block subsequent neurodegeneration
Early microglial activation and peripheral inflammation in DLB (33)	*In vivo* assessment of central and peripheral inflammatory changes in DLB patients using PET imaging with micro-glia activation marker ([^11^C](R)-PK11195-PET)	• Elevated microglia activation in several brain regions associated with cognitive functions • Microglial activation strongly correlates with cognitive score in DLB patients • Raised peripheral inflammatory cytokines
The peripheral inflammatory response to α-Syn and endotoxin in PD (34)	Investigation of cell-extrinsic factors in systemic immune activation by using α-Syn monomers and fibrils, as well as bacterial toxins, to stimulate both PD and control PBMCs	• α-Syn monomers or fibrils resulted in a robust cytokine response in both PD and control PBMCs
Increased immune activation by pathologic α-Syn in PD (35)	Investigation of immune response of primary human monocytes and a micro-glial cell line to pathologic forms of α-Syn	• Pathogenic α-Syn activates peripheral blood monocytes and microglial BV2 cell line leading to in-creased IL-6 release • Extracellular vesicles (EVs) from PD patient plasma induces a stronger activation peripheral blood monocyte than plasma EVs from healthy patients
Oligodendroglial α-synucleinopathy-driven neuroinflammation in MSA (36)	Analysis of temporal patterns of neuroinflammation in postmortem MSA-P brain and MBP29-hα-Syn mice	• Marked inflammatory myeloid response in corpus callosum and the striatum • Increased astrogliosis • Elevated CCL2, CCL7, and CXCL10 mRNA transcripts in α-Syn-overexpressing primary oligodendrocytes and MBP29-hα-Syn mice

*DLB, dementia with Lewy bodies; EVs, extracellular vesicles; PD, Parkinson's disease; MHCII, major histocompatibility complex class II; PET, positron emission tomography; D2R, dopamine D2 receptor; LPS, lipopolyschaccarides; BP, binding potential; PBMCs, peripheral blood mononuclear cells; MSA, multiple system atrophy; MBP, myelin basic protein; MBP29-hα-Syn mice, transgenic mice overexpressing human α-Syn under the control of an oligodendrocyte-specific MBP promoter; MCP-1, monocyte chemoattractant protein-1; MIP-1α/CCL3, macrophage inflammatory protein 1-alpha/Chemokine (C-C motif) ligand 3; RANTES/CCL5, regulated on activation, normal T cell expressed and secreted/chemokine (C-C motif) ligand 5; IL, interleukin; TNF-α, tumor necrosis factor-alpha; IFN-γ, interferon-gamma; SNpc, substantia nigra pars compacta*.

Mutant Htt is abundantly expressed in brain resident and peripheral immune cells, acting as an inflammatory stimulus for these cells (22, 23). In HD patients and several rodent models of HD, increased microglial activation, and elevated proinflammatory cytokines and chemokines have been shown to correlate with disease progression (19–21, 37–39). HD patients and several rodent models of HD show elevated plasma cytokine and chemokine expression levels (21, 22, 25). Mouse neuroblastoma cells expressing mutant Htt show an elevated level of chemokines, including monocyte chemoattractant protein-1 (MCP-1) and murine chemokine (40). NF-κB is a nuclear transcription factor and a key regulator of the inflammatory cascades. Activation of IκB kinases (IKK) leads to the release of NF-κB as a result of cytoplasmic sequestration by IκB and subsequent translocation to the nucleus. MHtt interacts with IκB, upregulating IKK, and NF-κB gene expressions in the immune cells of human HD and mouse models of HD (24, 41, 42).

Human α-Syn is a cytoplasmic protein with a 140 amino acid length encoded by the *SNCA* gene (43). It is mainly expressed in the presynaptic terminal in the central nervous system (CNS), but also partly found in the peripheral nervous system (PNS) and other tissues. Pathological α-Syn, secreted from neurons, interacts with glial cells to promote neuroinflammatory responses (30, 44). α-Syn activates innate and adaptive immune responses in several rodent models of PD and PD patients, as evidenced by pronounced neuroinflammatory changes within the brain as well as marked elevation of immune markers in the peripheral blood (29, 31, 32, 34, 45, 46).

In recent years, there is accumulating evidence that prionlike propagation of pathogenic proteins from cell-to-cell accounts for the progression of pathology in HD and α-synucleinopathies. These prion-like spreading and seeding capacities of pathogenic mHtt and a-Syn (either neuron-to neuron or neuron-to-glia) occur via several mechanisms, including exosomal transfer, synaptic transmission, and glial phagocytosis (47–54). Cell-to-cell transfer of pathogenic proteins disturbs neuroimmune network, leading to enhanced immune response and inflammation, as in these incurable neurodegenerative proteinopathies. Based on the notion that pathogenic protein aggregates lead to perturbed neuroimmune homeostatic network and consequent chronic immune activation, therapeutic strategies that aim to suppress immune activation or pathogenic proteins during neurodegenerative processes remain one of the popular treatment paradigms in several neurodegenerative disorders.

Over the years, several immunotherapeutic modalities for immunosuppression (using anti-inflammatory/antibody-based therapies), boosting the host's own adaptive immune response against specific pathogenic protein (via active vaccination) and/or antibody-mediated neutralization of specific pathogenic protein (passive vaccination) have been developed for HD and α-synucleinopathy. However, despite the remarkable recent progress, there remain intriguing concerns about the efficacy of these treatment modalities in human clinical trials.

In this article, we review several immunotherapeutic strategies that have been developed to suppress immune activation (using a variety of anti-inflammatory and antibody-based therapies) or eliminate pathogenic protein aggregates (via active and passive immunization) in preclinical rodent studies and human clinical trials for HD and α-synucleinopathies. We also discuss some of the pitfalls of single immunotherapy and place emphasis on the potential benefits of combination therapies or multi-target drugs in achieving successful clinical trials.

## Immunomodulatory Drugs for HD

Several preclinical and clinical trials of potential immunomodulatory drugs have been investigated in HD, such as laquinimod, anti-SEMA4D monoclonal antibody, and TNF-α inhibitors (Table 4).

**Table 4:** Clinical trial immunomodulatory agents and immunotherapies for HD and α-synucleinopathies.

**Drug and trial ID**	**Mechanisms of action**	**Trial phase/findings/status**	**References**
**Huntington's disease**			
Laquinimod (NCT02215616)	Anti-inflammatory Neuroprotection	Phase II safety and efficacy trial: Primary outcome not met but the secondary outcome of reduction of caudate atrophy was achieved Status: Completed	(55)
Anti-SEMA4D monoclonal antibody (VX15/2503-N-131) (NCT02481674)	CD100 antigen inhibitor Anti-inflammatory	Phase II: Safety, tolerability, pharmacokinetics, and pharmacodynamics, and efficacy trial Status: Ongoing till May 2020	(56)
Minocycline (NCT00029874) (NCT00277355)	Anti-inflammatory Caspase inhibition	Phase I/II/III: Well tolerated and safe. Phase III efficacy trial: Worsen disease progression as measured by a significant decline in total functional capacity, leading to trial futility. Status: Completed	(57, 58)
**Parkinson's disease and multiple system atrophy**			
PD01A and PD03A (NCT01568099) (NCT02618941) (NCT02267434)	Active immunization against α-Syn	Phase IA: Well tolerated and safe in healthy and early PD participants Status: Completed Phase IB follow-up Study: Well tolerated and safe. Significant increase of titers against PD01A, induction of PD01A-specific antibodies in CSF, and reduction of oligomeric and fibrillary α-Syn in plasma and CSF. Similarly, PD03A was well tolerated and showed a clear immune response against the peptide itself and aSyn targeted epitope Status: Completed	(59–61)
PD01A and PD03A (NCT02270489)	Active immunization against α-Syn	Parallel Phase I safety and efficacy trial in early MSA: Both drugs were safe, well-tolerated with clear immune response against the peptide itself and αSyn targeted epitope. PD03A, in contrast, showed no observable immune response compared to the placebo Status: Completed	(62)
MEDI1341 (NCT03272165)	Passive immunization against α-Syn	Phase I: Safety, tolerability, pharmacokinetics, and pharmacodynamics in healthy volunteers Status: Recruiting	(63)
PRX002 (NCT02095171) (NCT03100149)	Passive immunization against α-Syn	Phase I: Well tolerated and safe Status: Completed	(64, 65)
BIIB054 (NCT02459886) (NCT03716570)	Passive immunization against α-Syn	Phase1: Well tolerated and safe in healthy participants and early PD patients Status: Completed Phase I: Safety, tolerability, pharmacokinetics, and pharmacodynamics of BIIB054 in Japanese participants with PD are currently in progress Status: Recruiting	(66) (67)
Sargramostim (NCT01882010)	Immune modulator	Phase1: Well tolerated and safe in healthy participants and PD patients. Suppressed markers of immune activation in blood and improved cortical motor activity Status: Completed	(68)

### Laquinimod

Laquinimod is an orally active and well-tolerated immunomodulatory small molecule developed primarily to target inflammation and neurodegeneration. The efficacy of laquinimod has been evaluated for several clinical disorders including relapsing-remitting multiple sclerosis (RRMS), Guillain-Barré syndrome, Crohn's disease, lupus, and Huntington's disease (HD). Although the precise mechanism of action of laquinimod is unclear, evidence shows that it exerts both anti-inflammatory effects by driving Th (T helper cell) polarization from Th1-activating to Th2-activating cytokine production (69, 70) and as neuroprotective effects by promoting brain-derived neurotrophic factor (BDNF) production (71, 72). Gurevich et al. showed that laquinimod modulates antigen presentation-related genes and associated inflammatory molecules from peripheral blood mononuclear cells (PBMC) of RRMS patients (73). Dobson et al. demonstrated that laquinimod significantly dampened the release of hyper-reactive cytokines from stimulated premanifest and manifest HD patient monocytes (74). In YAC128 HD mice, laquinimod shows neuroprotective effects, rescuing corticostriatal neurodegeneration, white matter demyelination, and behavioral deficits (75, 76). Laquinimod ameliorates DNA-damage induced activation of caspase-6 by reducing Bax expression in primary neuronal cultures (77). Laquinimod provides a mild ameliorative effect on motor function deficit and striatal neuropathology in R6/2 HD mice (78). Recently, the efficacy of laquinimod (0.5 and 1.0 mg/d) in slowing down the progression of HD was evaluated in 352 HD patients using the Unified Huntington's Disease Rating Scale-Total Motor Score (UHDRS-TMS). In this study, laquinimod fails to meet its primary clinical endpoint of change (no significant difference in the primary efficacy outcome) for functional capacity in HD patients as assessed by UHDRS-TMS, from baseline, after 12 months of treatment. However, the secondary clinical endpoint of structural change was achieved, as revealed by a reduction of caudate atrophy (LEGATO-HD, ClinicalTrials.gov identifier NCT02215616).

These clinical findings raise the intriguing question of why the beneficial effect of laquinimod on caudate volume atrophy fails to impact on functional capacity (TMS and other clinical outcomes) in HD. One would expect a relevant correlation between MRI-detected structural brain changes and motor performance assessed by TMS. Overall, these findings indicate that reduction in motor function is not simply a footprint of regional brain volume abnormalities (striatal atrophy) but global structural brain network changes.

### Anti-semaphorin 4D

Semaphorin 4D (SEMA4D), otherwise known as CD100, is chemorepulsive axonal guidance and immunoregulatory transmembrane signaling molecule. It signals via three receptor subtypes, Plexin-B1 (PLXNB1), Plexin-B2 (PLXNB2), and CD72. PLXNB1 represents the high-affinity receptor of SEMA4D and is expressed in neurons, oligodendrocytes, endothelial cells, as well as some tumor cells (79–82). In the CNS, SEMA4D interacts with PLXNB1 in neuronal cells via Rho-GTPases-RhoA and R-Ras GTPase-activating protein activities, inducing axonal growth cone collapse (83–85). In the immune system, SEMA4D-CD72 interactions promote both B and dendritic cell activation, leading to T cell priming (86, 87).

Previous studies in rodents have demonstrated the efficacy of SEMA4D activity blockage in several disorders, including neurodegenerative diseases. Anti-Sema4D blocking antibody ameliorates neuroinflammation and development of experimental autoimmune encephalomyelitis (EAE) in mice (88). Similarly, PLXNB1 deficient mice are resistant to the development of EAE (88). Antibody neutralization of SEMA4D ameliorates neuropathological deficits and improves some of the behavioral symptoms in YAC128 HD transgenic mice (89).

Altogether, these findings support the immunomodulatory properties of SEMA4D in translational rodent models of inflammation-associated neurodegenerative diseases, including HD. Whether SEMA4D inhibition, however, will be beneficial in human HD is yet unknown. In 2015, Vaccinex Inc. in collaboration with the Huntington study group and the University of Rochester's clinical trials coordination center started the first clinical trial to investigate the safety, tolerability, and efficacy of Pepinemab, a humanized antiSEMA4D neutralizing monoclonal antibody (VX15/2503) as a potential treatment for late prodromal and early manifest HD patients, with specific focus on neuroimmunomodulation as well as delay of the onset and progression of HD. This study is currently ongoing with expected completion in May 2020.

### Anti-TNF-α Therapy

TNF-α is a multifunctional cytokine associated with cellular proliferation, differentiation, inflammation, immune responses, and apoptosis (90). TNF-α exists in 2 forms: a transmembrane stable homotrimeric form (tm-TNF-α), that is involved in immune functions by activating TNFR2, and a soluble homotrimeric form (sol-TNF-α), which mediates chronic inflammation by interacting with TNFR1, a death domain-containing protein (91–94). Tm-TNF-α is cleaved on the cell surface by a TNF-α-converting enzyme to yield the sol-TNF-α. The complex interaction between the death receptor (TNFR1) and death receptor ligands [sol-TNF-α or lymphotoxin (LT)-α] activates apoptotic signaling pathway through the help of adaptor proteins, such as Fas-associated protein with death domain, TNFR-associated death domain protein, and the TNFR-associated factor-1 (95). TNFR1 signaling complex recruits and dimerizes initiator caspase (caspase 8) which in turn, initiates apoptosis by cleaving and activating executioner caspases (caspase-3,-6, and-7) (96). Sol-TNF-α has been implicated as a hallmark of acute and chronic neuroinflammation and a key regulator of inflammatory responses in many neurodegenerative disorders, including MS, PD, AD, and HD. An elevated level of TNF-α in serum, cerebrospinal fluid (CSF), and brain tissue are associated with the pathophysiology of HD (21, 97) and a molecule that inhibits TNF-α signaling was shown to be beneficial in an HD rodent model (98). DN-TNF-α (XPro1595) is an inactive engineered human TNF-α variant that inactivates the native homotrimer sol-TNF-α via a subunit exchange mechanism, thus blocking TNF bioactivity (99). Hsiao et al. demonstrated that intracerebroventricular (ICV) infusion of DN-TNF-α modulates neuroinflammation, mHtt aggregate burden, caspase activation, and motor function deficit in R6/2 HD transgenic mice (98). It is worth noting that in comparison to ICV infusion, systemic injection of DN-TNF-α shows lesser efficacy on motor function in R6/2 mice. However, the clinical efficacy of this molecule in human HD warrants investigation. A most recent follow-up study by Pido-Lopez et al. demonstrated that systemic injection of etanercept, a TNFα inhibiting drug, dampens the plasma level of TNFα and other peripheral circulating proinflammatory cytokines such as IL1β and IL6 in preclinical HD R6/2 transgenic mice. Striatal TNFα and IL-6 expression levels, however, remain unaffected by etanercept treatment. In the same study, while treatment with etanercept partially reduces brain atrophy, it fails to ameliorate HD related functional and cognitive deficits in R6/2 mice (100). Lack of improvement in motor and cognitive impairment after etanercept treatment in R6/2 mice may be due to poor/low CNS distribution as etanercept is known to be BBB impenetrable (101). Therefore, further studies on central delivery of etanercept in HD preclinical mouse models might be warranted to validate etanercept efficacy. Minocycline, a second-generation tetracycline antibiotic, is another small molecule inhibitor of TNF-α signaling which decreases TNF-α synthesis, microgliosis, and TNF-α-induced caspase activation and apoptotic cell death (102). Minocycline has been shown to be protective in several neurological disorders, including PD, HD, amyotrophic lateral sclerosis, multiple sclerosis, stroke, and spinal cord injury (103). The impact of minocycline in the progression of HD symptoms has been previously assessed in various preclinical HD transgenic mice and HD patients. Preclinical evaluation of minocycline in HD raised many conflicting findings. Chen et al. and Stack et al. demonstrated that minocycline and combined minocycline and Coenzyme-Q10 therapy, respectively, delays disease progression and cell death in R6/2 HD mouse model (104, 105). Intriguingly, however, Smith et al. reported conflicting data, in which oral minocycline treatment in R6/2 mice shows no beneficial effects on behavioral abnormalities as well as mHtt aggregate load (106). The findings of Smith et al. were supported by several other studies in which minocycline showed a lack of efficacy in slowing the progressive functional decline in several HD mouse models (107–109). Also, in a phase III efficacy study (IND 60943), minocycline failed to show efficacy in HD patients (DOMINO Huntington study group, 2010). Taking together, these findings show that targeting peripheral circulating sol-TNFα helps to resolve neuroinflammation, but also suggest that suppressing neuroinflammatory process alone would be insufficient to restore functional capacity in HD.

## Immunotherapies for mHtt

### Active Immunization Against mHtt

Few studies have examined the beneficial effects of active vaccination in rodent models of HD, emphasizing the safety, immunogenicity, and efficacy of some vaccine immunogens. Ramsingh et al. examined the safety and immunogenicity of three non-overlapping Htt exon 1 peptides (AA1-17, AA4960, and AA74-88) in two different preclinical rodent models of HD (110). While the three peptides were safe, only AA1-17 induces stronger immunogenic response against Htt-targeted epitope in HD mutant mice. Vaccination using a combination of the three peptides showed stronger immunogenic response in both HD mutant and control mice. Notably, differential expression of immune-related genes, indicated by up-regulation of innate immune responses and downregulation of memory T cell responses were observed in immunized mutant HD mice (110). Additionally, DNA plasmid vaccination against mHtt ameliorates the diabetic phenotype in HD R6/2 transgenic mice (111). Studies of active immunization against mHtt remain obscure and warrant further follow-up preclinical studies to explicate immunogenicity, safety, and efficacy in different HD preclinical models.

### Passive Immunotherapy via Targeted mHtt-specific Intrabodies

**Figure 2:**
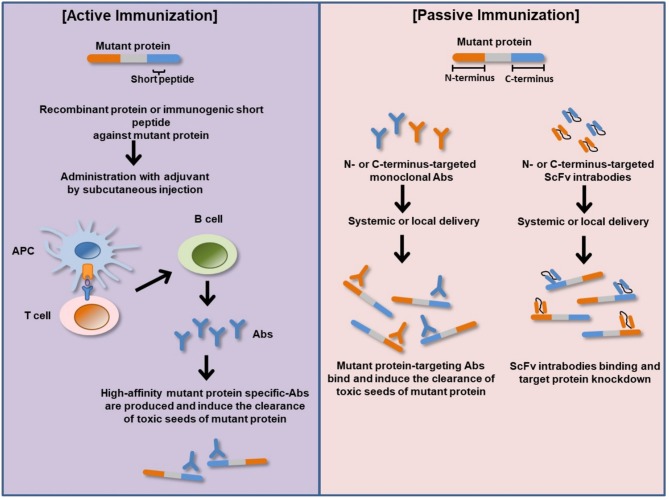
Basic principles of active and passive immunizations for mutant protein. In active immunization, a recombinant protein or immunogenic short peptide is administered to generate host immune response via plasma B-cells production of antigen specific-Abs that bind and eliminate their cognate targets (misfolded mutant protein). In passive immunization, exogenous recombinantly generated target specific-Abs or Ab fragments such as ScFv is administered to bind and neutralize their cognate target proteins within the body or cellular compartment. APC, antigen-presenting cell; Abs, antibodies; N-terminus, amino terminus; C-terminus, carboxyl terminus; ScFv, single-chain variable fragment antibody (intrabody).

Intrabodies are engineered single-chain variable fragments antibodies (scFv) expressed inside cells and directed to different subcellular compartments to bind different epitopes on a target protein (Figure 2) (112). They act as effector molecules with a capacity to neutralize or sequester specific proteins in a particular cellular compartment. ScFv intrabody consists of variable heavy chain (VH) and variable light-chain (VL) domains joined by a flexible polypeptide linker (112). Several studies have analyzed the therapeutic potential of several recombinant anti-Htt-directed intrabodies to specifically counteract the downstream intracellular mHtt pathologic cascades in cell culture and preclinical rodent models of HD. The intrastriatal fusion of recombinant adeno-associated virus (rAAV)-expressing anti-N-terminal Htt-exon 1-scFv-C4 intrabody results in delay accumulation of mutant Htt in HD R6/1 transgenic mice (113, 114). Similarly, enhancement of intrabody's efficacy via fusion of the C-terminal PEST region of mouse ornithine decarboxylase (MODC) to scFv-C4 significantly reduces mHtt aggregation and toxicity compared to scFv-C4 alone in a striatal cell line expressing exon 1 of mHtt with 72 glutamine repeats (113). ScFv intrabodies against N terminus and proline-rich (PRR) domains of Htt (VL12.3 and Happ1, respectively) reduce mHtt-induced toxicity and aggregation in an *in vitro* and corticostriatal brain slice models of HD (115). Concordantly, rAAV-expressing Happ1 ameliorates the neuropathology and prolongs survival in four different HD transgenic mice (116). Transduction with rAAV-expressing INT41, intrabody specific for the PRR domain of Htt, significantly reduces mHtt aggregate loads and ameliorates cognitive decline in HD R6/2 transgenic mice (117). Recombinant AAV-expressed scFv-EM-48 suppresses mHtt accumulation and ameliorates neuronal dysfunction in R6/2 and N171-82Q transgenic mice (118).

Together, the findings support the therapeutic relevance of passive immunization via targeted mHtt-specific intrabodies in preclinical HD models. However, the translational prospect in terms of safety, tolerability, and efficacy in human HD remains undetermined.

### Immunomodulatory Drugs for α-synucleinopathies

Recent studies have identified many potent immunomodulatory agents, including sargramostim and azathioprine (Table 4). Suppressing the peripheral immune response might offer an effective disease-modifying strategy for PD patients.

### Sargramostim

Sargramostim, otherwise known as leukine, is a recombinant human granulocyte macrophage colony-stimulating factor (GM-CSF). GM-CSF is a potent immune modulator known to promotes regulatory T cells (Treg) activities and dampen pro-inflammatory T effector (Teff) responses, thereby protecting against inflammation-mediated neurodegeneration. The homeostatic balance between circulating T cell subsets is impaired in several rodent models of PD and PD patients (119). In fact, T cells from PD patients recognize α-Syn peptides as antigenic epitopes (120). In a randomized, placebo-controlled double-blind phase 1 trial in PD patients and non-Parkinsonian subjects, treatment with sargramostim was feasible and reasonably well-tolerated (68). More so, sargramostim improves magnetoencephalography-recorded cortical motor activities and immunomodulatory activities of Tregs (68). However, the therapeutic potential of sargramostim warrants a larger cohort and further confirmatory studies.

### Azathioprine

Azathioprine is an inosine monophosphate dehydrogenase (IMDH) inhibitor with an immunosuppressive function. Azathioprine treatment is associated with a lower risk of PD (121). Azathioprine inhibits IMDH which leads to the blockage of DNA synthesis and consequent suppression of circulating T-cell activity. Since T cells from PD patients recognize α-Syn peptides as antigenic epitopes, the suppressive activity of T-cells might dampen peripheral propagation of α-Syn. Just like sargramostim, the therapeutic potential of azathioprine as an immunosuppressant for PD is promising but warrants a larger cohort and further clinical studies. Also, whether the long-term use of azathioprine, like other immunosuppressants, such as corticosteroids, will engender serious potential morbidity such as cardiovascular disease, metabolic dysfunction, osteoporosis, remains unknown.

### Immunotherapies for α-Syn Pathology

A vast number of clinical trial potential α-Syn immunotherapies have been investigated in α-synucleinopathies, including active and passive immunization against α-Syn and immunomodulatory drugs (Figure 2 and Table 4).

### Active Immunization Against α-Syn

The potency of active immunization targeting α-Syn has been examined in a Lewy Body (LB) disease mouse model. In this study, transgenic mice overexpressing human wildtype α-Syn under control of the platelet-derived growth factor-β promoter was used. These mice present α-Syn aggregation in neurons of the cortex, hippocampus, and olfactory bulb (122). When mice were immunized with recombinant human α-Syn, high-affinity α-Syn antibodies directed to its C-terminus were produced. The antibody-treated mice exhibited a reduction of α-Syn accumulation in neuronal cell bodies and synapses, leading to amelioration of neurodegeneration. Importantly, in both adjuvant-treated and human α-Syn-vaccinated animals, there was a mild neuroinflammatory response as demonstrated by the microglial marker (Iba1) and the astroglial marker [glial fibrillary acidic protein (GFAP)]. However, no differences in Iba1 and GFAP markers were detected in both groups, suggesting that microglia might not be involved in the clearance of αSyn. Notably, antibodies produced by immunized mice were targeted to the C-terminal region of α-Syn with higher affinity. Consequentially, short epitopes of α-Syn were developed for inducing α-Syn-targeting antibodies.

Ghochikyan et al. generated three peptide-based epitope vaccines composed of α-Syn-derived short peptides fused with a T helper epitope from tetanus toxin (P30) (123). Immunization of mice with these vaccines generated high titers of anti-α-Syn that can bind to LBs in brain tissues from DLB patients.

Dendritic cells (DCs) play an important role in initiating primary immune responses, through the antigen presentation to T cells (124). DC-based vaccination appears to be one of the cell-based therapeutic strategies to elicit an immune response using human α-Syn-sensitized DCs. Based on this strategy, Ugen et al. developed fragments and full-length human α-Syn protein-sensitized bone marrow-derived DCs to generate vaccine for PD (125). Sensitized DCs-injected transgenic mice expressing human A53T variant α-Syn showed significant improvement of locomotor function without an inflammatory response.

Nevertheless, it would be important to perform further studies in detail for the mechanisms before moving the above strategies to clinical trials.

### Short Peptides-AFFITOPEs^®^ (AFF1)

AFFITOPEs^®^AFF1 is a C-terminal α-Syn mimicking small molecule and peptide developed by the Austrian pharmaceutical company AFFiRiS AG. This immunogenic peptide is too short to cause α-Syn-specific T cell and autoimmune response. Active immunization with AFF1 resulted in decreased accumulation of α-Syn oligomers and reduced degeneration of tyrosine hydroxylase (TH) positive fibers in the caudo-putamen regions in PDGF-and mThy1 promoter controlled α-Syn transgenic mice for PD and DLB, respectively (122).

Furthermore, AFF1 vaccination increased the clearance of α-Syn via microglial activation and the production of anti-inflammatory cytokines such as IL-1Ra, IL-2, and IL-27. In a mouse model of MSA, AFF1 treatment diminished the spreading of α-Syn from oligodendrocytes to astroglial cells and alleviated demyelination and neurodegeneration in the neocortex, striatum, and corpus callosum (126). More importantly, repeated immunization led to the recovery of motor function, learning, and memory. It is suggested that clearance of α-Syn involved activation of microglia and reduced spreading of α-Syn to astroglial cells leads to improvement of locomotor- and cognitive function. Phase 1 testing is currently in preparation for the following two AFFITOPE^®^ vaccines that target α-Syn using MSA as a model for α-synucleinopathies.

### PD01A and PD03A

Based on the beneficial effects in preclinical research, including animal experiments, AFF1 progressed to human clinical trials as AFFITOPEs^®^PD01A and PD03A, synthetic peptides against αSyn. PD01A treatment in early-stage PD patients showed local and systemic tolerance without any severe or unexpected reactions. In another study, a different synthetic epitope of α-Syn, PD03A was tested. This trial was a randomized, placebo-controlled, parallel-group, patient-blind, two-center study, assessing tolerability, and safety of repeated subcutaneous administration of two different doses of PD03A to patients with early PD. Both doses of PD03A were locally and systemically well-tolerated with no unexpected effect.

A parallel phase 1 clinical trial (NCT02270489) evaluated a high-dose of PD01A and PD03A in 30 MSA patients over 36 weeks. PD01A and PD03A were well-tolerated in early MSA patients. Interestingly, PD03A showed a clear dose-dependent immune response against the peptide itself and cross-reactivity against the α-Syn targeted epitope over time while PD03A showed no significant immune response compared to placebo.

Although the clinical studies were not set up or powered to properly estimate the efficacy, PD01A is intended to investigate the efficacy in PD patients in a phase II trial in the second half of 2020.

### Passive Immunization Against α-Syn

Passive immunization for PD is performed by using a monoclonal antibody targeting α-Syn. The reason for choosing passive immunization rather than active immunization is to reduce the dose and avoid the potential side effects. Passive immunization has the potential to target and induce the clearance of toxic seeds of α-Syn, including aggregated and fibrillary forms in neuronal populations and the propagation (Figure 2).

There are two main targets for monoclonal antibodies for αSyn in C-terminal and N-terminal regions. C-terminal α-Syn is implicated to play an important role in the pathogenic properties in PD (127, 128). A mouse monoclonal antibody against the C-terminus of α-Syn (9E4) (epitope aa 118–126) significantly decreased the C-terminally truncated α-Syn aggregates in axons and synaptic terminals and improved motor and cognitive deficits in PDGF-α-Syn transgenic mice (129). Another laboratory showed similar results of motor behavioral improvement and reduction in α-Syn following treatment with C-terminal antibody AB274 (epitope aa 120–140, mIgG2a) in the same transgenic mice (130). Interestingly, the uptake of Ab274/α-Syn complex by microglia was observed, suggesting that the degradation of α-Syn may be primarily by microglia.

Three other antibodies, 1H7 (epitope aa 91–99), 5C1, and 5D12 (epitope aa 118–126) were also tested in Thy1 promoter-controlled human α-Syn transgenic mice (131). All these antibodies showed some beneficial effects on PD motor behavior deficits and neuropathology Several N-terminal-directed antibodies against α-Syn were also developed and tested in various rodent models of PD. Treatment with N-terminal anti-α-Syn antibody (Syn303) (epitope aa 1–5) ameliorated the spread of α-Syn aggregates and the loss of dopaminergic neurons, as well as improved locomotor functions in intrastriatal α-Syn pre-formed fibrils (PFF)-injected mice (132). Also, N-terminal antibody (AB1) (epitope aa 16–35) treatment in an AAV-α-Syn rat model of PD resulted in moderate motoric improvement with the concomitant rescue of the substantia nigra dopaminergic neurons as well as modulation of microglial activation (133). Due to the pathogenicity of α-Syn in its aggregated conformational states, the use of antibodies with special properties for targeting oligomers and fibrils have been suggested and documented. Immunization with a monoclonal antibody that targeted oligomeric/prefibrillary forms of α-Syn (mAb47) mitigates the accumulation of oligomeric αSyn in the brain stem and improved motor behavioral performance (134). Recently, Kallab et al. showed three antibodies (Syn-F1, Syn-O1, and Syn-O4) that specifically target oligomeric α-Syn and damped motor behavior deficit and neuroinflammation (135).

Overall, passive immunization using a targeted α-Syn antibody is quite promising for modifying disease pathology in rodent models of PD. These findings have been advanced into human clinical trials, evaluating the safety and tolerability in healthy volunteers.

### PRX002

The first candidate of passive immunization for PD patients was PRX002, a humanized version of the 9E4 antibody, developed by Prothena Biosciences. PRX002 administration in several doses showed good tolerability, favorable safety, and pharmacokinetic profiles with no immunogenicity in PD patients (64). Serum level of free α-Syn was significantly reduced following PRX002 administration, whereas total α-Syn increased, dose-dependently, because of the expected change in kinetics following the binding of the antibody. More recently, a trial of multiple ascending doses of PRX002 was performed in patients with idiopathic PD (65). In this randomized clinical trial, single and multiple ascending doses of PRX002 were generally safe and well-tolerated and resulted in robust binding of peripheral αSyn. Also, PRX002 cerebrospinal fluid levels increased in a dose-dependent manner following treatment, with concentrations that may be expected to engage extracellular aggregated α-Syn in the brain. In 2017, a phase II multinational study of PRX002/RO7046015 in newly diagnosed PD patients was initiated in collaboration with F. Hoffmann-La Roche AG (PASADENA Study, ClinicalTrials.gov identifier NCT03100149).

### BIIB054

BIIB054 developed by Biogen Inc. (rights to BIIB054 were acquired from Neuroimmune AG) is the second antibody going into a clinical trial. BIIB054 is a fully human IgG1 monoclonal antibody directed at the N-terminus of α-Syn and is highly selective for the aggregated forms of α-Syn with 800-fold higher apparent affinity for fibrillary vs. monomeric α-Syn (136). In a randomized phase I clinical trial, BIIB054 showed favorable safety, tolerability, and pharmacokinetic profiles in volunteers and PD participants. All PD participants showed almost complete saturation of the BIIB054/α-Syn complex formation, indicating that it binds much less of its monomeric form. Currently, BIIB054 is being studied in the ongoing phase II trial (SPARK study, ClinicalTrials.gov identifier NCT03318523).

### MEDI1341

MEDI1341 is a potent α-Syn C-terminal specific synthetic human monoclonal antibody co-developed by AstraZeneca plc and Takeda Pharmaceutical Company Ltd. It binds human, cynomolgus monkey and rat α-Syn monomers and aggregates. Recent preclinical study shows that MEDI1341 blocks cell-to-cell spread of pathogenic α-Syn in an *in vitro* human SH-SY5Y-donor cells pretreated with pre-sonicated human preformed α-Syn fibrils (137). Also, systemic administration of MEDI1341 to rat and monkeys achieves high brain penetration and suppresses extracellular α-Syn in the CNS as evidenced by significant reduction in the CSF and brain interstitial fluid α-Syn levels (137). Of interest, passive immunization with either MEDI1341 or its engineered effector null mutant version MEDI1341-D265A mitigates hippocampal and neocortical α-Syn expression as well as transhippocampal tracts/axons spreading of α-Syn in mice injected with lentiviral vector expressing human-α-Syn (137). Evaluation of MEDI1341 for safety, tolerability, pharmacokinetics, and efficacy is currently in human clinical testing (ClinicalTrials.gov identifier NCT03272165).

## Future Perspectives

**Figure 3:**
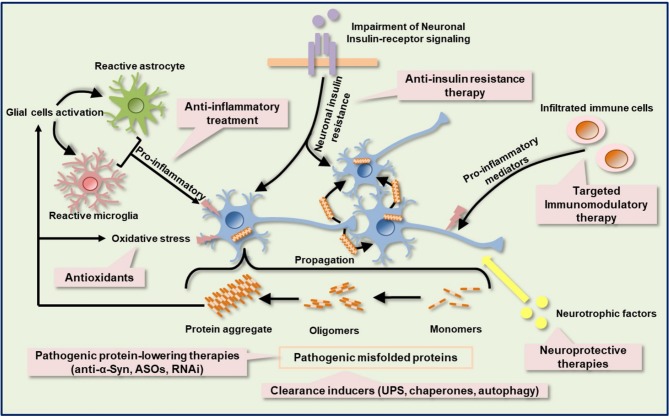
Schematic of the targeted immune-based therapies with other possible neuroprotective and neurorestorative strategies. Protein aggregation, neuroinflammation, oxidative stress, metabolic and mitochondrial deficits, insulin signaling impairment, and neurotrophic factors dysregulation are complex components of the pathologic cascade in several neurodegenerative diseases, including HD and PD. Immunotherapy against pathogenic protein can suppress mutated protein aggregation. Activation of protein quality control machinery, such as chaperones, autophagy, and UPS facilitates the clearance of misfolded proteins. Other misfolded protein-lowering therapy includes ASOs and RNAi. Anti-inflammatory agents help to ameliorate neuroinflammation by dampening the release of proinflammatory molecules. Selective blockage of leukocytes infiltration into the CNS can also be achieved by targeted immunomodulatory therapy. Impairment of insulin signaling contributes to metabolic dysfunction and anti-insulin resistant therapy may ameliorate metabolic dysfunction and confer a neuroprotective effect. Neurotrophic factors protect and repair damaged neurons. ASOs, antisense oligonucleotides; RNAi, RNA interference; UPS, ubiquitin proteasome system.

Despite the advancement in the development of immune based therapies for HD and α-synucleinopathies, alleviation of disease progression has not been fully achieved. Most of the immunotherapies and anti-inflammatory agents currently used to treat a variety of neurodegenerative diseases have little to no disease-alleviating properties, which accounts for their failures in clinical trials. It appears that the pitfalls of these compounds are in the multiple pathogenic cascades associated with these neurodegenerative diseases. Intuitively, the human brain is a very complex system and sometimes the structural measures do not necessarily predict functional capacity (i.e., correspond to function). In other words, one cannot account for functional and cognitive recoveries by looking at the structural changes alone, particularly when damage is not restricted to a single brain region, as is the case of proteinopathy-induced neurodegenerative diseases. Targeting the immune system might be insufficient to restore functional capacity in HD and α-synucleinopathies. Also, since neurodegenerative diseases show complexity, combination therapies or multi-target drugs targeting several dysregulated mechanistic pathways might be required to achieve a significant functional recovery and halt or delay disease progression (Figure 3). Combination therapy is a multi-modality therapeutic approach targeting multiple components of biological regulatory circuits in order to achieve stronger efficacy in terms of addictive or even synergistic effects (138, 139). According to a mathematic model, combination therapies that target distinct pathways might produce a stronger, long-lasting treatment efficacy for a disease than individual treatments (140, 141). By identifying candidate drugs that target distinct pathways and administering them in combination may offer long-lasting beneficial effects for neurodegenerative proteinopathies. Taking a lesson from cancer immunotherapy, amalgamation of anticancer drugs that target key pathways in a characteristically synergistic or addictive manner proffers enhanced efficacy and efficiency compared to the monotherapy approach (142). In preclinical and clinical studies in which either cancer monotherapy targeting program cell death protein 1 (PD-1) or costimulatory receptor, glucocorticoid-induced tumor necrosis factor receptor-related protein (GITR) showed limited efficacy in mouse cancer models (143, 144), combination therapy achieved synergistic effects, leading to stronger T-cells activation and enhanced tumor control in mouse cancer models (145–147). Also, the efficacy of the anti-SEMA4D antibody as an immunomodulatory therapy in tumors was enhanced by combination with other immunotherapies, including anti–CTLA-4, anti–PD-1, and cyclophosphamide (81, 82).

Although therapeutic targets against α-Syn are beneficial in cellular and rodent models of PD (130, 135), their translational efficacy in human clinical trials still poses a major challenge as underlying mechanisms are not fully understood.

Relatedly, anti-inflammatory agents currently used to treat a variety of neurodegenerative diseases demonstrated efficacy in various rodent preclinical models of HD; however, disease alleviating efficacy has rarely been achieved in clinical trials (55, 57).

Dysfunction of protein quality control systems such as autophagy, the ubiquitin-proteasome system, and chaperones have been reported in HD and α-synucleinopathies (148, 149) Enhancement of mHtt and α-Syn degradation through the above systems or some enzymes, neurosin (kallikrein 6) (150) may support the clearance of pathological α-Syn. After neural damage, several neurotrophic factors such as brain-derived neurotrophic factor (BDNF), ciliary neurotrophic factor (CNTF), glial cell line-derived neurotrophic factor (GDNF), cerebral dopamine neurotrophic factor (CDNF), and vascular endothelial growth factor (VEGF), which provide neuroprotective and neurorestorative effects via anti-inflammatory, anti-apoptotic, re-myelination, and axon regeneration properties, are significantly altered (151). VEGF has shown apparent neuroprotective effects in rodent preclinical models of PD, which is accompanied by an improvement in motor symptoms (152). Moreover, the use of VEGF in combination with other neurotrophic factors has shown a synergistic effect in 6-hydroxydopamine (6OHDA) partially lesioned rats (153). Additionally, Valera et al. demonstrated the stronger efficacy of combination of αSyn-targeted passive immunotherapy (CD5-D5) and anti-inflammatory treatment (lenalidomide) over each single treatment in MBP-α-Syn transgenic mice (154), further strengthening the therapeutic potential of strategic combination of treatments in neurodegenerative proteinopathies. Metabolic dysfunction such as type II diabetes mellitus has been associated with HD and PD. The mechanisms by which metabolic dysfunction induces exacerbation of neurodegenerative diseases are still not clear. Intuitively, neurodegenerative diseases and type II diabetes mellitus show similar pathologies, including mitochondrial impairment, oxidative stress, and chronic inflammation. Interestingly, insulin signaling and related molecule pathways were disturbed in neurodegenerative diseases and type II diabetes mellitus (155). Insulin signaling is known to be essential for neuron survival (156–158).

Based on these findings, targeted multi-modality therapy could serve as a potential treatment strategy for polyQ diseases and α-synucleinopathies, where several potential immunotherapies have failed as disease-slowing treatments. The potential efficacy of these novel drugs might be enhanced through a combined therapy with other potential targeted drugs, such as mutant protein lowering agents (antisense oligonucleotide, RNAi, anti-α-Syn), chaperones, neurotrophic factors, anti-insulin resistance therapy, and antioxidants. Remarkably, early diagnosis and prompt treatment intervention during premanifest disease stage are crucial for successful combination treatment regimens in clinical trials.

## Summary and Conclusion

Neuroinflammation is a major contributing factor in several neurological disorders, validating the use of immunotherapies and anti-inflammatory agents, such as anti-inflammatory cytokine biologics, as a therapeutic option to alleviate disease burden. In this review, we summarized findings evaluating the beneficial effects of immunotherapies in HD and α-synucleinopathies. Immunotherapies and anti-inflammatory agents have rarely achieved robust effectiveness in alleviating HD and α-synucleinopathies in human clinical trials. Evaluating the combined treatment strategy of immunotherapy with multiple target neurorestorative candidate drugs to target different processes that complement each other or the same process at different levels of pathogenesis for additive or synergistic effects, could probably show better efficacy in slowing down disease progression. While targeted multi-modality therapy is promising in terms of greater clinical efficacy, it is limited by its potential side effects and toxicity. Therefore, as with all monotherapy development, it is important to understand the molecular mechanisms, possible drug-drug interaction, and tolerability of side effects (safety) of each mono-drug and combination therapy. Also, optimizing the dose of drugs in combination, by using a lower dose, might help to reduce the risk of toxicity and side effects.

The development of multimodality therapy that targets inflammation-mediated progression and neurocircuitry disruptions might be warranted to alleviate disease burden as well as to improve the duration and quality of life of patients suffering from proteinopathy-induced neurodegeneration such as HD, SCAs, PD, DLB,b and MSA.

## Author Contributions

All authors listed have made a substantial, direct and intellectual contribution to the work, and approved it for publication.

### Conflict of Interest

The authors declare that the research was conducted in the absence of any commercial or financial relationships that could be construed as a potential conflict of interest.

## Abbreviations

HD: Huntington's disease
PD: Parkinson's disease
mHtt: mutant Htt protein
DLB: dementia with Lewy bodies
MSA: multiple system atrophy
SCAs: spinocerebellar ataxias
α-Syn: α-synuclein
mAb: monoclonal antibody
UHDRS-TMS: Unified Huntington's Disease Rating Scale-Total Motor Score
IL: interleukin
IT-15: interesting transcript 15
SCNA: α-synuclein gene
LB: Lewy body
LN: Lewy neurite
BBB: blood-brain barrier
MMP: matrix metalloproteinase
TNF-α: tumor necrosis factor-alpha
IFN-γ: interferon-gamma
C1q: complement component 1q
CXCLs: chemokine (C-X-C motif) ligand 1
iNOS: inducible nitric oxide synthase
ROS: reactive oxygen species
RNS: reactive nitrogen species
APC: antigen-presenting cell
Abs: antibodies
N-terminus: amino terminus
C-terminus: carboxyl terminus
ASOs: antisense oligonucleotides
RNAi: RNA interference
UPS: ubiquitin proteasome system.
